# Milk production status and associated factors among indigenous dairy cows in Raya Kobo district, north eastern Ethiopia

**DOI:** 10.1002/vms3.740

**Published:** 2022-01-26

**Authors:** Silamlak Birhanu Abegaz

**Affiliations:** ^1^ Department of Biology Faculty of Natural and Computational Sciences Woldia University Woldia Ethiopia

**Keywords:** Ethiopia, indigenous dairy cows, milk production status, Raya Kobo

## Abstract

**Background:**

A cross‐sectional survey study was conducted from September 2020 to April 2021. A total of 217 households were randomly selected. The data collection instruments were structured questionnaires: focus group discussion and key informant interviews. Data were coded, entered and analyzed using Statistical Package for the Social Sciences (SPSS) version 20 software. Ranking indexes as well as binary logistic regression analysis were used to look for the relationship between dependent and independent variables.

**Result:**

The present study showed that season of calving, disease and parasite challenges, housing conditions and shortage of land for forage production with an index value of 0.180, 0.154, 0.153 and 0.126, respectively, were the most important constraints affecting milk production potential. Likewise, foot and mouth disease [adjusted odds ratio (AOR) = 0.001, 95% confidence interval (CI) = (0.000–0.016)], internal parasites [AOR = 0.003, 95% CI = (0.000–0.046)], shortage of grazing land [AOR = 0.017, 95% CI = (0.002–0.148)], summer season of calving [AOR = 0.012, 95% CI = (0.002–0.088)], overall cattle herd composition [AOR = 0.002, 95% CI = (0.000–0.025)], straw shed [AOR = 0.046, 95% CI = (0.006–0.327)] and open yard [AOR = 0.003, 95% CI = (0.000–0.183)] housing conditions were significantly associated with milk production status at *p* < 0.001 and *p* < 0.05.

**Conclusion:**

The current study indicated that milk production status was poor. Therefore, suitable government policy support and provision of subsidies, genuine participation of dairy producers with governmental and non‐governmental organizations are imperative to improve livestock productivity. Furthermore, future research and development actions should find solutions to decrease the bottlenecks so that the massive potentials of the area could be exploited to its maximum and could advance the livelihood of the community.

## Graphical abstract

Milk productivity status among indigenous dairy herds was poor in Raya Kobo district. Season of calving, disease and parasite challenges, housing condition, shortage of land for forage production and cattle herd compositions were the significant constraints influencing dairy production in the study area.

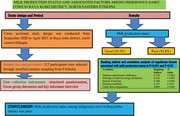

## 1 BACKGROUND

Livestock husbandry plays a substantial role in the economy of nearly all regions in Africa. It represents on average 20%–40% of agricultural gross domestic product (GDP) (Negassa et al., 2013). Ethiopia has the highest record of livestock in Africa. However, its productivity and maximum financial gain remain low. In Ethiopia, the main milk production sources were cows which supply 83.4% of the total yearly milk yield of the country (FAO, 1993). But slight amounts of milk are also acquired from goat and camel in animal farming areas of the country (Negassa et al., 2013). Latest investigations confirmed that the government of Ethiopia has underestimated the impact of ruminants in the whole value contribution of agriculture sector. However, the dairy sub‐sector alone contributes 63% to the overall value of ruminant output (Chanyalew, 2017). Furthermore, current figures point out that the livestock sector subsidizes about 12%–16% of national GDP, 30%–35% of agricultural GDP, 15% of export wages and 30% of agricultural employment (Ali, 2013). At present, livestock farming is undervalued or less supported by the government and non‐government organizations. Thus, the development of this sub‐sector is facing lack of focus. Of course, smallholder farmers are representatives of 98% milk production in Ethiopia. However, productivity is still quite low, support services are inadequate, and quality feeds are challenging to get.

In Ethiopia, the development of genetic improvement programmes for livestock dairy production was started since the period of Italian occupation. However, these interventions have been met with little achievement for the reason that a number of practical, structural and socio‐economic limitations were existing (Yilma et al., 2011). Simultaneous cattle breed improvement and milk production success are complemented when there was a better understanding of various constraints including feeding, housing, health control and management (Ruben et al., 2017). There was a dairy product insufficiency in all pastoral areas of Ethiopia. Nevertheless, the tendencies of economic assessments for dairy industry attainment and progress are slightly improved both at small scale and commercial levels of the country (Chebo & Alemayehu, 2012). There are several factors influencing milk production potential of dairy cows in many parts of Ethiopia including Raya Kobo district. According to the study of Getabalew et al. (2019), shortage of land for grazing and cultivation of improved forage, diseases and parasites, poor level of performance of cattle, inadequate veterinary service and shortage of labour and artificial insemination (AI) service were existing constraints of dairy production in Ethiopia. In addition, absence of permanent trade routes, lack of transport, inadequate infrastructural and institutional set‐ups and poor market information (inside and outside the country) are generally stated as some of the most significant reasons for the reduced enactment of this sector (Kidanu, 2010).

In the study area, marketing, processing, transportation and administration of milk were major challenges for dairy farming communities and varied from one location to another. Even, livestock facilities, such as feed, natural/AI services, immunization and de‐worming, are time‐sensitive; however, these facilities have been failed to distribute equally in a timely manner owing to logistical, financial and management limitations. In order to lessen the above‐mentioned factors, growing the proficiency of AI deliverance, upgrading veterinary vaccinations and introducing better quality forage crops and feed trees are imperative (Yami et al., 2013). Depending on their localities, dairy farming practices are divided into three comprehensive classes in Ethiopia; namely, urban, peri‐urban and rural dairy farming system (Galmessa et al., 2013). In the study area, among the prevailing dairy farming systems, the rural dairy farming system is the one that is persistently existing and commonly practiced. Pastoral dairy farming system is one of the sustenance farming practices that promotes up to 98% of the entire milk manufacture in Ethiopia, and includes pastoralists, agro‐pastoralists and mixed crop‐livestock manufacturers (Guadu & Abebaw, 2016). The traditional (smallholder) dairy farming system, which is dominated by indigenous (local) breeds, added up to 97%–98% of the whole annual milk yield in Ethiopia (Bereda et al., 2014). However, over 85% of the milk manufactured by pastoral household is utilized within the pastoral families with the proportion being marketed less than 7% due to its greatest benefit for children's general well‐being, strength and development (Gobena, 2016). In the study area, milk manufacturing is a routine activity, for sale or cash and procurement of processed outputs. Dairying is a cash crop in the milk‐shed areas that allows people to purchase additional products and considerably supports the families food security (Nyekanyeka, 2011). Milk and its outputs are economically significant farm supplies, and livestock farming is a good investment option as it plays a major role in feeding pastoral as well as non‐pastoral communities in Ethiopia. However, milk manufacturing trends in Raya Kobo district was very poor, and most of the milk manufactured is only for home feasting. The level of excess milk is determined by the potential to produce milk in terms of herd size and production season. The collected milk is mainly processed using traditional skills and the processed milk outputs such as butter, ghee, ayib and sour milk are commonly sold through the low‐priced market after the households meet their requirements (Tekea, 2021). Promoting and marketing of dairy farming could have an imperative role in changing the livelihoods of families and grow up incomes among small holders in Raya Kobo district. However, essential interferences and design of policies are still lacking in order to elevate small‐scale dairy farming year after year. The low productivity in dairy sector is due to many factors mentioned for the existing problems in the country. Furthermore, in‐depth identification of those problems in the sector regarding dairy productivity, management and health issue is important. Those identified problems correlated with distinct types of production scale are also important to tackle the existing problems in the dairy farm sector. Therefore, knowing the putative factors may help to conduct a wide range of research and provide important information for policy makers so as to review the quality, quantity and economic consequences of milk due to these factors and to give more emphasis on salient amelioration strategies to counter the adverse impact of the existing environmental factors particularly on milk somatic cell counts (SCCs) and reduce milk losses in the study area. Thus, the present study aims to assess milk production status and associated factors among indigenous dairy cows in Raya Kobo district.

## 2 MATERIALS AND METHODS

### 2.1 Description of the study area

**FIGURE 1:**
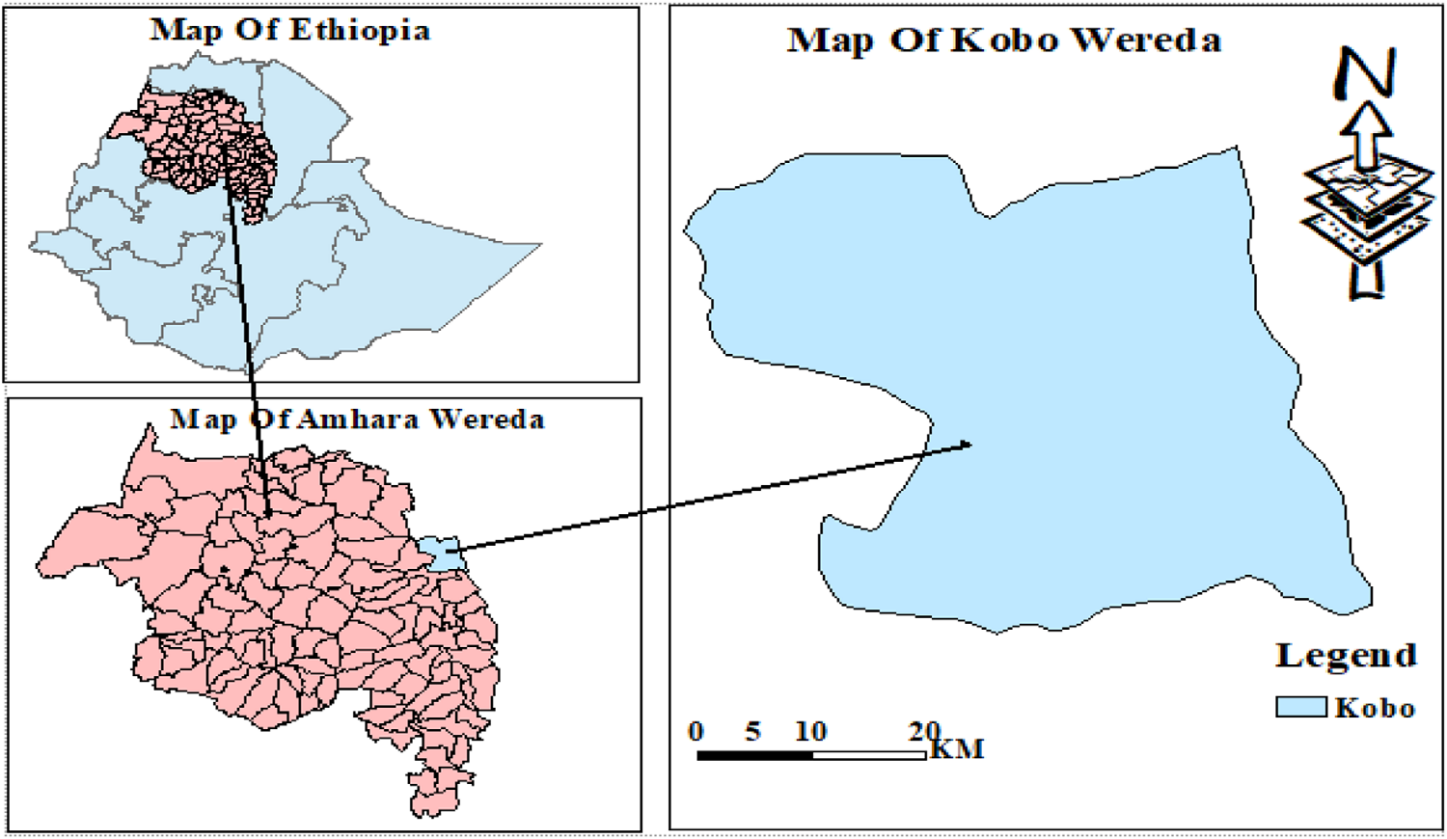
Map of the study area. *Source*: www.div‐agis/gdat

Kobo or Ray Kobo district (Figure 1) is located in the northeast of Amhara Regional state of Ethiopia. Raya Kobo or Kobo district is at distance of 579 km from Addis Ababa, the capital of Ethiopia. According to the Central Statistical Agency of Ethiopia (CSA, 2007), this district has a total of 221,958 residents, of these inhabitants 111,605 are males and 110,353 are females. The woreda is characterized by three different agro‐ecological zones: highland, midland and lowland, and having bimodal rainfall patterns. The chief raining period of the district starts from June to September although short rainy period is very common from March to May. The district obtains a higher and lower amount of 800 and 500 mm rainfall per year, respectively, having yearly highest and lowest temperature of 33 and 12°C (Derbie et al., 2019).

### 2.2 Research design

A cross‐sectional survey study was conducted from September 2020 to April 2021 in Raya Kobo district, Northeast Ethiopia.

### 2.3 Study population

All the households that live in Raya Kobo district who were aged 18 and above were included in this study.

### 2.4 Sample size determination

Regardless of its agro‐climatic zones, within the district there are forty‐five kebeles; however, only nine kebeles have dairy farming potential (Derbie et al., 2019). The selected kebeles also represented the highland, midland and lowland agro‐climatic zones found in the district. Therefore, these kebeles were nominated purposively for this study. Then, the necessary household sample size was allocated to assess the current status of milk production in Raya Kobo district. Therefore, sample size determination was made using the statistical method of Cochran (Equation 1), assuming a non‐response rate of 5% and design effect of 1.5 (Cochran, 1977):

(1) $$n=\frac{\left[z^{2}\times \left(P\times \left(1-P\right)\right)\right]}{d^{2}}\mathrm{thus},n=\frac{{\left(1.96\right)}^{2}\times \left(0.10\right)\times \left(1-0.10\right)}{{\left(0.05\right)}^{2}}=138$$

where *n* is sample size of study subjects, *z* is standardized normal variable and its value corresponds to a 95% confidence interval (CI) equals 1.96, *d* is allowable error (0.05) and *P* is 0.10 (10%) for the proportion of farmers expected to achieve good milk production status. Adding 5% for non‐responses and multiplying the sample size by 1.5 for design effect, the sample size was added up to 217 study participants (Cochran, 1977).

### 2.5 Sampling technique

**FIGURE 2:**
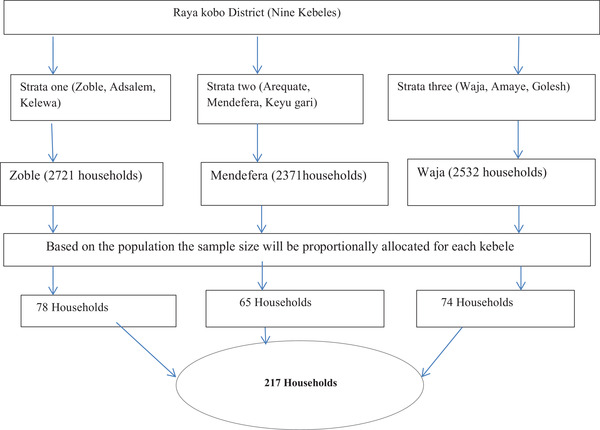
Schematic representation of sampling procedure

For participants’ selection, based on geographical location of the district, the nine kebeles were divided into three strata. A kebele is the smallest administrative unit in Ethiopia, similar to a ward or a neighbourhood. Next, one kebele was selected from each stratum using the random sampling technique. Finally, participants were selected proportionally from the three kebeles based on their population size and samples were drawn randomly (Figure 2).

[Correction added on 1 February 2022, after first online publication: In Section 2.5 “nine kebeles” was corrected to “three kebeles” in this version]

### 2.6 Data collection instruments

Structured and standardized survey questionnaires were adapted from Bereda et al. (2014). The questionnaires were used to assess the status of milk production and related factors among indigenous dairy cows in the study area. And questionnaires further developed by reviewing the literature and relevant factors such as housing conditions of dairy cows, disease control, cows feed, year of calving, season of calving, age within parity and volume of milk produced at different stages of lactation per cow/day and so forth were taken into consideration (Bereda et al., 2014). Questions were translated into the local language (Amharic) by language specialists. The questionnaire included both close‐ and open‐ended questions, socio‐demographic factors and several other factors associated with milk production. Household heads who manage family members were eligible for the questionnaire survey. Illiterate respondents were assisted by questionnaire administrators.

### 2.7 Focus group discussion and key informant interviews

Focus group discussion was also used to collect data from 35–40 farmers grouped into three focus group discussions, each containing 8–13 individuals. Focus group participants were a small, cautiously designated group and equal in social construction. The local administrators and development agents working in the Woreda office of agriculture and rural kebeles were encompassed and helped in identifying the names of the focused group in which different religion, age, gender, classes and educational levels were considered. Issues related to factors affecting milk production status among indigenous dairy cows were discussed in each focus group session. In addition, key informant interviews (farmers suggested by the agricultural community for their outstanding indigenous technical knowledge on cattle breeding, dairy farming, managing and consumption) were employed to complement the information obtained from individual farmers as well as focused group discussions. Focused group discussions and key informant interviews were conducted during the main reproduction season from September 2020 to April 2021.

### 2.8 Study variables

Milk production status (good or poor) was the dependent variable, whereas average milk production at different lactation stages per cow/day, inadequate AI services, shortage of land for forage production, housing conditions of dairy cows, disease and parasite challenges, cows feed, year of calving, season of calving, age within parity and herd size and composition and so forth were the independent variables.

### 2.9 Data analysis

The measurable data were analyzed by means of descriptive statistics, and the qualitative data were analyzed by narration. Data were first checked manually for completeness and then coded, entered and analyzed using Statistical Package for the Social Sciences (SPSS) version 20 software. Moreover, an index was calculated to provide overall ranking of dairy production constraints in the study area by developing rank index formula based on the method used by Amare et al. (2019) and Dinku (2019). That is, $\frac{(Rn\;\times \;C1\;+\;Rn\;-\;1\;\times \;C2\;\text{…}.\;\;+\;R1\;\times \;Cn)\;a\;-\;g}{\sum_{a\;-\;g\;}\left(Rn\;\times \;C1\;+\;Rn\;-\;1\;\times \;C2\;\text{…}.\;+R1\;\times \;Cn\right)}$ where, *Rn* is value of the last rank of constraint *a* (if the last rank is 14th, then *Rn* = 14, *Rn* −1 = 13, *R*1 = 1), *Cn* is counted value of the last rank level (in the above example, the counts of the 14th rank = *Cn*, and *C*1 is the count of the 1st rank) and (*Rn* × *C*1 + *Rn* − 1 × *C*2 …. + *R*1 × *Cn*) *a* − *g* is weighted summation of each constraints (a, b, c … g). Finally, bivariable and multivariable logistic regression analysis were used to look for the relationship between the dependent and independent variables. Independent variables in the bivariate logistic regression model with a *p*‐value < 0.25 were incorporated in the multivariable logistic regression.

### 2.10 Operational definition

Milk production status: Milk production potential of dairy herds under considerable environmental circumstances.

Good milk production status: The average peak milk production potential of dairy cows was relatively better or close to the national average 1.54 L per cow per day over a lactation period of 180 days (Getabalew et al., 2019; Tesfaye et al., 2010).

Poor milk production status: The average peak milk production potential of dairy cows was relatively lower to the national average 1.54 L per cow per day over a lactation period of 180 days (Getabalew et al., 2019; Tesfaye et al., 2010).

### 2.11 Limitation of the study

The cross‐sectional nature of the study design limits the applicability of the findings in establishing causality between the variables.

## 3 RESULTS

### 3.1 Household characteristics at different sites included in the study

The inhabitants of the study areas have different religion (in decreasing order of origin), age group (mostly 41–50 on average) and educational level (mostly they are illiterate and literate). The economies in the study area were principally depending on rain‐fed subsistence cultivation of mixed crops and livestock production (Table 1). The farmers in the study area are mainly involved in small‐scale agriculture, often using flood irrigation with floods from the escarpment. In recent years, farmers have started dry season irrigation agriculture, encouraged by government founded ground water pumps and by imitating profit‐making farms that have been attracted.

**TABLE 1:** Household characteristics at different sites of Raya Kobo district included in the study from September 2020 to April 2021 (*n* = 217)

Age groups		Educational status		Gender		Average family size	Average farm size (ha)	Major crops and livestock
18–30	31	Illiterate	104	Male	120	4.6	0.51	Sorghum, teff, maize, wheat, cattle, Small ruminant, donkey
31–40	74	literate	57	Female	97			
41–50	83	Primary education	35					
>50	29	Secondary education	21					
		Tertiary education	–					

*Source*: Local farmers and Woreda agricultural office during survey time 2020/2021.

### 3.2 Milk production status in Raya Kobo district

#### 3.2.1 Farmers’ response in relation to milk production status and trends in Raya Kobo district

A total of 217 farmers took part in the study providing 100% response rate. This response rate (100%) was attained because the data gathering processes were intelligently carried out. In addition, data organizers were skilled, and the issues of milk production practice were not complex. As a result, the respondents were not failing to obey with the envisioned study. Eighty‐five (39.2%) of the farmers stated that a form of butter milk was utilized for home consumption only, preliminary by children in the family than any other member. In addition, 101(46.5%) of the farmers replied that the produced milk was utilized for sale and home consumption purposes. Eighty‐three (38.2%) of the farmers responded that 1–2 L (average) of milk was produced at different lactation stages per cow per day. Overall, herds are mainly composed of lactating cows followed by heifers and calves which account nearly three‐fourths of the animals in the herd. The main purpose of rearing livestock in the district is milk and meat production (Table 2).

**TABLE 2:** Distribution of farmers citing milk production status and trends in Raya Kobo district from September 2020 to April 2021 (*n* = 217)

Variables	Category	Frequency	Percent
What is the milk production status in the study area?	Good	98	45.2
	Poor	119	54.8
Form of milk more consumed	Uncooked state	68	31.3
	Fermented milk	64	29.5
	Butter milk	85	39.2
Purpose of milk production	Home consumption only	116	53.5
	Sale and home consumption	101	46.5
Priority of milk consumption in the family	Husband	76	35.0
	Children	100	46.1
	Wife	41	18.9
Average milk production at different lactation stages per cow/day	1–2 L	83	38.2
	3–4 L	69	31.8
	>5 L	65	30.0
Overall cattle herd structure in Raya Kobo district	Lactating cow	49	22.6
	Dry cow	40	18.4
	Heifer	48	22.1
	Calves	48	22.1
	Bull	32	14.7
Main purposes of livestock rearing in Raya Kobo district	Milk production	74	34.1
	Meat production	61	28.1
	Income generation	49	22.6
	Others	33	15.2

*Source*: Local farmers and Woreda agricultural office during survey time 2020/2021.

#### 3.2.2 Reasons for good/poor milk production status in Raya Kobo district

**FIGURE 3:**
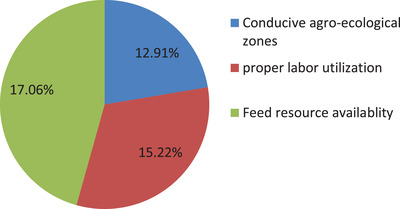
Reasons for good production status of milk among indigenous cows in Raya Kobo district, Northeastern Ethiopia. *Source*: Local farmers and Woreda agricultural office during survey time 2020/2021

**FIGURE 4:**
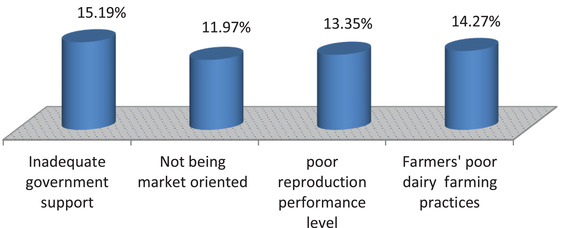
Reasons for poor production status of milk among indigenous cows in Raya Kobo district, Northeastern Ethiopia. ***Source***: Local farmers and Woreda agricultural office during survey time 2020/2021.

From 98 farmers, about 37(17.06%) of them cited feed resource availability as the topmost reason for good production status of milk (Figure 3), whereas of 119 farmers, 33 (15.19%) cited inadequate government support as the main reason for poor production status of milk in the study area (Figure 4).

#### 3.2.3 Major constraints of dairy production development in the study area

Milk production development in the study area was affected by a number of factors. The most important constraints associated with milk production in the study area were prioritized by the respondents and indicated in Table 3. There was a variation in index intensity among constraints limiting dairy production in Raya Kobo district. Season of calving, disease and parasite challenges, housing conditions and shortage of land for forage production with an index value of 0.180, 0.154, 0.153 and 0.126, respectively, were the most important constraints affecting milk production potential (Table 3).

**TABLE 3:** Dairy production constraints ranked by the respondents and priority indexes in the study area from September 2020 to April 2021 (*n* = 217)

	Weighted frequency									
Description	1	2	3	4	5	6	7	8	Index	Rank
Inadequate artificial insemination	56	22	43	23	17	12	34	19	0.109	6th
Disease and parasite challenge	51	73	63	18	30	47	–	7	0.154	2nd
Shortage of land for forage production	58	12	39	63	21	30	27	29	0.126	4th
Year of calving	13	59	23	45	29	37	40	77	0.118	5th
Season of calving	75	81	45	–	43	66	59	–	0.180	1st
Age within parity	33	24	14	11	–	–	49	98	0.071	8th
Overall cattle herd composition	4	15	69	9	29	35	12	68	0.085	7th
Housing conditions	59	74	33	46	24	43	–	–	0.153	3rd

*Source*: Output from survey data, 2020/2021.

#### 3.2.4 Association of major constraints with milk production status in the study area

The relationship of each independent variable with the status of milk production was verified using binary logistic regression analysis. In the multivariate analysis, foot and mouth disease, internal parasites, shortage of grazing land, summer season of calving, overall cattle herd composition, straw shed and open yard housing conditions were significantly associated with milk production status at *p* < 0.001 and *p* < 0.05 (Table 4).

**TABLE 4:** Bivariate and multivariate analysis of associated factors with milk production status among indigenous dairy cows in Raya Kobo district Northeastern Ethiopia from September 2020 to April 2021

	Milk production status		OR (95% CI)			
Variables	Good *n* (%)	Poor *n* (%)	COR (95% CI)	*p*‐Value	AOR (95% CI)	*p*‐Value
Inadequate artificial insemination						
Yes	51 (23.5)	61 (28.1)	0.969 (0.568–1.655)	0.909	2.170 (0.423–11.134)	0.353
No	47 (21.7)	58 (26.7)	1		1	
Disease and parasite challenges						
Foot and mouth disease	38 (17.5)	22 (10.1)	0.232 (0.111–0.484)	0.000**	0.001 (0.000–0.016)	0.000**
Internal parasites	40 (18.4)	47 (21.7)	0.470 (0.241–0.917)	0.027*	0.003 (0.000–0.046)	0.000**
External parasites	20 (9.2)	50 (23.0)	1		1	
Shortage of land for forage production						
Homestead land (ha)	25 (11.5)	33 (15.2)	1		1	
Cropping land (ha)	23 (10.6)	36 (16.6)	1.186 (0.567–2.479)	0.651	2.504 (0.417–15.049)	0.316
Grazing land (ha)	35 (16.1)	15 (6.9)	0.325 (0.146–0.721)	0.006*	0.017 (0.002–0.148)	0.000**
Forage land (ha)	15 (6.9)	35 (16.1)	1.768 (0.796–3.924)	0.161	0.287 (0.036–2.267)	0.237
Year of calving						
1–4	17 (7.8)	58 (26.7)	1		1	
5–10	52 (24.0)	37 (17.1)	0.209 (0.105–0.414)	0.000**	0.109 (0.010–1.243)	0.074
>10	29 (13.4)	24 (11.1)	0.243 (0.113–0.521)	0.000**	0.442 (0.034–5.725)	0.532
Season of calving						
Winter	29 (13.4)	74 (34.1)	1		1	
Summer	69 (31.8)	45 (20.7)	0.256 (0.144–0.452)	0.000**	0.012 (0.002–0.088)	0.000**
Age within parity						
0 and 1st	27 (12.4)	30 (13.8)	1		1	
2nd and 3rd	29 (13.4)	33 (15.2)	1.024 (0.498–2.105)	0.948	1.129 (0.110–11.592)	0.919
4th and 5th	42 (19.4)	56 (25.8)	1.200 (0.623–2.313)	0.586	0.258 (0.050–1.320)	0.104
Does overall cattle herd composition affect milk production in the study area?						
Yes	64 (29.5)	55 (25.3)	0.457 (0.263–0.792)	0.005*	0.002 (0.000–0.025)	0.000**
No	34 (15.7)	64 (29.5)	1		1	
Housing condition						
Tin shed	35 (16.1)	31 (14.3)	1		1	
Straw shed	36 (16.6)	35 (16.1)	1.098 (0.561–2.147)	0.785	0.046 (0.006–0.327)	0.002*
Open yard	27 (12.4)	53 (24.4)	2.216 (1.134–4.330)	0.020*	0.003 (0.000–0.183)	0.006*

Abbreviations: AOR, adjusted odds ratio; CI, confidence interval; COR, crude odds ratio; OR, odds ratio.

Statistically significant at: ** = *p* < 0.001, * = *p* < 0.05

*Source*: Output from survey data, 2020/2021.

## 4 DISCUSSIONS

The present study was undertaken to determine milk production status and associated factors affecting dairy yield in Raya Kobo district. Milk is the most important livestock output made by smallholder crop‐livestock farmers and most of the pastoral households who keep cow in order to produce milk for family consumption and income. The indigenous dairy cows play a significant role in maintaining a strong agricultural economy of Raya Kobo Woreda. Dairy farming can play a leading role in reducing undernourishment of the study area, particularly for children. However, several factors limit the production potential of these cows. According to Nuri (2019), dairy farming is commerce, a means of living and year round job. Dairy business is somewhat money‐making, and pastoral communities have plenty of chances to upsurge the productivity by using more of collective feed and hired labour inputs (Quddus, 2018). The present study confirmed that different forms of milk were consumed in the study area; however, butter milk shares the highest proportion. In addition, in the study area, the primacy of milk intake in the nutrition is largely accepted, and it has a very high recurrence of demand as compared to other foods. The economics of dairy production can be made more commercial by upgrading the production potential of dairy cows through breeding programmes. Improvement of dairy sub‐sector may be helpful and a significant approach for poverty mitigation which could be a major objective of the government (Shiferaw et al., 2011). In the study area, the average milk production at different lactation stages per cow/day/household was 1–2 L (Table 2). On the contrary, the average daily milk yields of local cows were 3.4 ± 0.9, 2.8 ± 1 and 0.9 ± 0.6 L in early, mid and late lactation stages, respectively, across production systems in West Gojam (Gizaw et al., 2017). In addition, in the Ezha districts of Gurage Zone, the overall average daily milk production per cow per household was 1.83 ± 0.08 L (Dinku, 2019).

These figures were also thought to be relatively better than the national average 1.54 L per cow per day over a lactation period of 180 days (Getabalew et al., 2019; Tesfaye et al., 2010). The discrepancies between reports could be due to study area settings and existing environmental constraints. In agreement with the study of Derbie et al. (2019), in the present study, the total number of milk‐producing cows and calves possessed per household was relatively higher compared to bulls (Table 2). The rational was farmers are committed to harvest more milk for their family consumption and income. According to the study of Beriso et al. (2015), there are 87,771 and 18,760 cattle and goats in Chuko woreda, respectively. In Raya Kobo district, the major dedications of nurturing livestock are to increase milk and meat production that afford the livelihood of the families.

Dairy improvement in emerging nations have played a big part in growing milk production, scale up income level in rural areas, creating employment options and transforming the food security of the societies, especially for small holders and marginal farmers (Adesogan & Dahl, 2020). Even though, dairy production have been a means of promoting year‐round working alternatives for households to utilize their family labour effectively, provision of support and marketing systems remain weakening (Ahmed & Kobayashi, 2013). In the study area, none is practiced towards milk production as market oriented, government supported and institutionalized manner. Generally, according to the studies of Amare et al. (2019) and Dinku (2019), the scope and variety of livestock assets have become vital to sustenance of life in rural community and particularly, the largely agricultural economy of Ethiopia. Therefore, the cattle establish the leading component of livestock treasure in Ethiopia both in the agrarian high lands and pastoral and agro‐pastoral low lands, and therefore the proportionate impact on the country's economy could be high.

A number of factors have been limiting milk production potential of Raya Kobo district. For that reason, season of calving, disease and parasite challenges, housing conditions and shortage of land for forage production were most importantly prioritized constraints by farming communities. Their corresponding index values were 0.180, 0.154, 0.153 and 0.126. The study conducted by Bereda et al. (2014) pointed out that lack of land (45%), shortage of feeds (41%) and AI services (10%) were reported to be the major constraints to the milk production in Southern Ethiopia. Furthermore, the study of Amare et al. (2019) showed that land shortage, feed shortage and labour were the first three constraints in large‐scale dairy production with an index value of 0.43, 0.35 and 0.19, respectively. AI services (0.06 for medium‐scale dairy production, 0.08 for small‐scale dairy production) and diseases (0.04 for medium‐scale dairy production, 0.05 for small‐scale dairy production) were also the most important constraints affecting dairy production in Ethiopia (Amare et al., 2019). The difference in index value might be because of study area setting, respondents’ demographic characteristics and exiting environmental factors. Housing was ranked as the primary problem in dairy herds due to lack of sufficient space for each group of animals based on age category and production (De Vries et al., 2015). Higher proportions (79.28%) of small‐scale dairy farms were managed in traditional free stall compared to large‐ and medium‐scale farms in Ethiopia, and only 11.71% of the dairy farms were managed under modern barn without individual cattle pen (Amare et al., 2019). Likewise, these factors including foot and mouth disease [adjusted odds ratio (AOR) = 0.001, 95% CI = (0.000–0.016)], internal parasites [AOR = 0.003, 95% CI = (0.000–0.046)], shortage of grazing land [AOR = 0.017, 95% CI = (0.002–0.148)], summer season of calving [AOR = 0.012, 95% CI = (0.002–0.088)], overall cattle herd composition [AOR = 0.002, 95% CI = (0.000–0.025)], straw shed [AOR = 0.046, 95% CI = (0.006–0.327)] and open yard [AOR = 0.003, 95% CI = (0.000–0.183)] housing conditions were significantly associated with milk production status at *p* < .001 and *p* < .05 (Table 3). In agreement with the current study, the study conducted in Afar region pointed out that foot and mouth disease whose odds ratio found between 15.8 and 23.79 at 95% CI was the most prevalent and economically significant disease in the study region and about 19.8% and 56.94% of the disease was estimated during the study period (2018–2019) at animal and herd level, respectively (Dubie & Negash, 2021). The possible explanation could be Raya Kobo district shares common national boundaries with Afar region and due to highly contagious, disease of all cloven‐hoofed animals and transboundary nature of the disease may attribute to its prevalence. Livestock diseases can result in economic losses to the pastoral dairy farming system in Ethiopia, accounting for hundreds of millions of birr every year (Jemberu et al., 2016). These diseases are currently widespread in all geographical areas of the country and annual mortality rates because these diseases are estimated to be 8%–10% for cattle herds, 15% and 12% for sheep and goat flocks, respectively (Dubie & Negash, 2021). It is expected that livestock diseases decrease production and productivity of livestock approximately by 50%–60% per year (Ganeshkumar, 2012). The current study also revealed that indigenous dairy cows were 99.7% less likely affected by internal parasites compared to external ones. Internal parasites can exist as long as cattle are grazing pastures. However, the severity of infection will vary with age and stress level of the animal (Afolabi et al., 2017). Parasite burdens are most detrimental in mature cows near parturition because immunity is suppressed. Particularly, dairy cows in early lactation are often in a negative energy balance due to lactation stress, hence reducing milk production, weight gain and conception rate (Sheldon et al., 2019). Moreover, the gastrointestinal parasites such as *paramphistomum* following *Fasciola* and *Ascaris* are the most common parasitic worms of concern in Raya Kobo district. The intensity of parasite pressure in a pasture varies with season and management. Parasite pressure could be higher during the spring and is lower during the hot, dry summer months (Rose et al., 2016). Parasite burden could also be lower under good management conditions. Shortage of grazing land was another factor which is 0.017 times influencing milk production status in the study area compared to homestead land. It is very common that rangelands were largely utilized for livestock production development. However, its sustainability is threatened by unmanageable land‐use systems and continuous grazing (Espeland et al., 2020). Thus, milk yield gain could be affected in the study area. According to the study of Lalampaa et al. (2016), the average milk yields (106 ± 20.1) of animals in holistic grazing areas were significantly (*p* < 0.05) higher than those in traditional grazing areas (101 ± 20.1). In the study area, the findings of the present study showed that season of calving particularly summer season less likely affected milk production status by 98.8% compared to winter season. Dairy production development systems worldwide are typically vulnerable to extreme environmental and seasonal fluctuations such as heat stress (Polsky & von Keyserlingk, 2017). Milk somatic cells (SCs) are a mixture of milk‐producing cells and immune cells. These cells are secreted in milk during the normal course of milking and are used as an index for estimating mammary health and milk quality of dairy animals worldwide (Alhussien & Dang, 2018, 2017). Environmental stresses such as extreme temperature and humidity intensely increase the amount of SC found in milk. The higher SCCs in milk, the lower the products with a shorter shelf life and vice versa. As the SCC rises, the severity of intramammary infection elevates and leads to a decreased milk yield and quality (Mukherjee et al., 2015). levels of SCC during hot‐humid season become higher in elite cows compared to non‐elite cows indicating more stress level on the udder of these animals during this particular season (Mukherjee et al., 2015). In milk, casein (except γ‐CN) was lower in the summer and higher during the winter season, whereas immunoglobulin G and serum albumin contents were higher in summer than in winter and spring seasons (Bernabucci et al., 2015). Milk coagulation properties were worsened during the summer season. Values of milk SCC and neutrophil:macrophage (N:M) ratio were highest during the summer season, lowest during thermo neutral and intermediate in winter (Alhussien & Dang, 2018, 2017). Pathogens usually enter the quarter through the teat canal before, during and after lactation periods. During dry periods and between milking, teat canal is sealed by a keratin plug which is an effective physical and microbicidal barrier against invading microorganisms. However, damage to the plug can temporarily or permanently increase the penetrability of the teat canal, thus increasing the chances of bacterial growth, replication and then mammary infections (mastitis). This leads to an increase in white blood cells in milk and indicates poor hygiene of the produced milk (Alhussien & Dang, 2018, 2017). The study participants reported that opportunistic pathogens such as *Streptococcus uberis, Enterococcus* spp., *Arcanobacterium pyogenes*, coagulase‐negative *Staphylococci* and coliforms were the most common mastitis pathogens prevalent in dairy herds of the study district than any other contagious pathogens. High intensity of heat stress due to increasing air temperature and humidity could also be difficult for cows to cool themselves (Alhussien & Dang, 2018). Therefore, managing high intensity of heat stress demands a high energy cost for affected dairy cows and leads to altered metabolism, hormone and feed intake rates. This in turn can lead to reductions in milk production (Alhussien & Dang, 2018, 2017). As milk production cools in the summer, farmers try to provide fodder for cows to generate heat. The way cows digest food takes a lot of energy and generates a lot of heat. This makes them lose their appetite and produce less milk (Alhussien & Dang, 2018, 2017). The Intergovernmental Panel on Climate Change (IPCC) has indicated that the earth's atmospheric temperature has already risen by 1°C (Masson‐Delmotte et al., 2018) compared to pre‐industrial time, and this global trend is likely to continue with 1.5°C warming (in total) in the next 30 years. The number of heat waves has increased alongside their duration and intensity, and it is predicted that an increase of 0.2°C per decade is likely to occur (Islam, 2021; Masson‐Delmotte et al., 2018). Collectively, the outcome of this climate change has increased animal heat stress events, impacting productivity (Key et al., 2014), welfare (Polsky & von Keyserlingk, 2017), sustainability (Das et al., 2016) and the viability (Gaughan et al., 2010) of the cattle industry.

In the present study, presence of overall cattle herd composition was another significant factor influencing milk production potential by 99.8% compared to their counterparts. Similar investigations confirmed that with the increase in overall herd size and composition, visual and clinical monitoring for every individual would not be practical (Barriuso et al., 2018; Gaughan et al., 2010). Moreover, controlling transmission of zoonotic disease could be more difficult among dairy cows in the herd and obstructs milk production (Megersa et al., 2011).

Another factor, housing condition in the present study has also shown a significant relationship with milk production status at *p* < 0.05. That is, open yard and straw shed were found to be less likely affect milk production status by 99.7% and 95.4%, respectively, compared to tin shed (Table 3). The yard and sheds in the study area were characterized by low and poorly drained ground, less protective from sunlight, storms and cold winds and also very poor sanitary conditions. The environment in which dairy cows spend the majority of their time has considerable impact on productivity, health, milk quality, reproduction, animal well‐being and farm profitability (Bewley et al., 2017). Housing of dairy cows is critical for monitoring of animals, dairy farming and disease control. But housing construction requires a substantial cost and effort. In fact, the smallholder farmers can invest manual labour and built less expensive cowsheds (Quddus, 2018). A number of scholars indicated that various environmental constraints can affect dairy productivity. According to Engdaw (2015) lack of grazing land, communicable and parasitic diseases, housing and scarcity of land for cultivation of better‐quality forage, inadequate vaccination, and low milk production performance of local cattle, insufficient AI services and labour shortage were the major challenges affecting milk production capabilities in pastoral community. Another study by Beriso et al. (2015) showed that the majority of agrarians stated that shortage of grazing land was the main problem of milk production followed by disease and parasites in Ethiopia. In addition, inadequate feeding both in quality and quantity, housing, shortage of breeding bull or AI service, poor veterinary services, unavailability of better genotypes and phenotypes of native animals that are actually mentioned worth are reflected in low milk production and dairy product marketing (Getachew & Tadele 2015). The study conducted by Dereje et al. (2015) revealed that the difference in average daily milk production between the study sites may be due to differences in management, extent of nutrition and type of breed found in North and South Wollo zones.

## 5 CONCLUSION

Even though livestock production development plays a significant role in the economy of pastoral communities in Raya Kobo district, its production status and commercialization remain low. For the appropriate intervention to be implemented, understanding the production status of milk asset is a pre‐requisite. The present study indicates that the productivity of dairy cattle is limited by several constraints including season of calving, disease and parasite challenges, housing conditions and shortage of land for forage production were the most importantly prioritized constraints by farming communities. Their corresponding index values were 0.180, 0.154, 0.153 and 0.126. Likewise, these factors including foot and mouth disease, internal parasites, shortage of grazing land, summer season of calving, overall cattle herd composition, straw shed and open yard housing conditions were significantly associated with milk production status at *p* < 0.001 and *p* < 0.05 (Table 3). The constraints encountered in this study were due to the lack of suitable policy support and provision of subsidies from the government. Therefore, governmental and non‐governmental organizations should participate genuinely for the sustainable use of the dairy cattle, and dairy producers should also be trained on various aspects of improving dairy cattle productivity (nutritional, health, housing and breeding management, etc.) and develop their entrepreneurial skills. Furthermore, future research and development endeavours should find solutions to curtail the bottlenecks of this sector so that the vast potentials of the area could be exploited to its maximum and improve the livelihood of the community.

## CONFLICT OF INTEREST

The author declares no conflict of interest.

## AUTHOR CONTRIBUTIONS

SBA contributed to conceptualization of research idea, designing and data collection and analysis, data interpretation, searching resources, writing – original draft, review & editing, read and approve the final manuscript.

## ETHICS STATEMENT

The study was approved by the Institutional Research Ethics Review committee (IRERC) of Woldia University after a review of the research protocol. The respondents/households/ were informed about the purpose of the study, and their oral consent was obtained. The respondents’ right to refuse or withdraw from partaking was fully retained and the evidence given by each participant was saved strictly private. Before entering the study area to collect data, local authorities, Woreda agricultural office heads and community leaders were informed about the objective of the study.

### PEER REVIEW

The peer review history for this article is available at https://publons.com/publon/10.1002/vms3.740

## Data Availability

The data used to support the findings of this study are included within the article.
